# A Feasibility Study of a Controlled Standing Fulcrum Side-Bending Test in Adolescent Idiopathic Scoliosis

**DOI:** 10.3390/jcm13247809

**Published:** 2024-12-20

**Authors:** Christian Wong, Christos Koutras, Hamed Shayestehpour, Benny Dahl, Miguel A. Otaduya, John Rasmussen

**Affiliations:** 1 Department of Orthopedic Surgery, Rigshospitalet, 2100 Osterbroo, Denmark; 2 Department of Orthopedic Surgery, Copenhagen University Hospital, 2650 Hvidovre, Denmark; benny.dahl@regionh.dk; 3 Department of Computer Science, Universidad Rey Juan Carlos, 28942 Madrid, Spain; christos.koutras@urjc.es (C.K.); miguel.otaduy@urjc.es (M.A.O.); 4 Department of Materials and Production, Aalborg University, 9220 Aalborg, Denmark; hs@anybodytech.com (H.S.); jr@mp.aau.dk (J.R.)

**Keywords:** adolescent idiopathic scoliosis, spine flexibility test, quantitative force measurements, fulcrum side-bending test

## Abstract

**Background/Objectives**: Spinal flexibility radiographs are important in adolescent idiopathic scoliosis (AIS) for clinical decision-making. In this study, we introduce a new method, the ‘quantitatively controlled standing fulcrum side-bending’ test (CSFS test). This is a feasibility study; we aimed to quantify the applied force and track the temporospatial changes in the spine specifically by measuring the continuous change in the Cobb angle (in degrees) during lateral bending. **Methods**: In this cross-sectional study, we included patients with AIS. Using a low-dose cinematic fluoroscopic technique, we captured the lateral bending of the thoracolumbar vertebral spine while inducing quantified lateral force on the ribs by a force gauge in a three-point fixation setup of controlled lateral bending. Trial registration number: H-1703423. **Results**: Twenty-one patients with small-curve AIS were included as subjects. All subjects performed the CSFS test adequately. They had small curves with a mean Cobb angle of 12.0 (range: 0.0–26.0, SD: 7.1). The measured median stiffness was 3.66 N/degrees (°) of the Cobb angle (range: 0.02–11.81) with a median coefficient of determination R^2^ of 0.58 (range: 0.002–0.92) by regression analyses. When analysed concerning the median-term clinical outcome of either progression/regression or stationary curves, various Cobb angle measurements and the other experimental parameters, there were no significant relationships. **Conclusions**: The CSFS test is feasible to quantify the force applied and the temporospatial changes in the spine during lateral bending. The CSFS test has been utilised in basic research for mechanical characterisation of the scoliotic spine and has the potential of being implemented directly in patient-specific bracing to estimate the forces needed for brace correction and adjustment so as not to supersede the allowed skin pressure.

## 1. Introduction

Several spinal flexibility tests have been introduced. These entail either separate or a combination of lateral- or fulcrum-bending or traction methods of the spine performed while supine or in the standing position [1]. They have been examined in relation to the risk of progress and as a prognosticator for bracing and surgical outcomes for adolescent idiopathic scoliosis (AIS) [2,3,4,5]. Five types of flexibility methods have been utilised previously, namely the supine side-bending method (the supine method), the fulcrum-bending method (the lateral bending method), traction, the push-prone method (the manual correction method and combined with traction), and the suspension method (the traction method) [1]. The supine side-bending method is the most commonly used, and the fulcrum-bending method is the most accurate method in terms of estimating the immediate and final follow-up postoperative corrective outcome; however, there is diversified reporting about which flexibility method provides the best estimation of postoperative outcomes [5]. Flexibility rates of more than 28% have been linked to an increased likelihood of preventing curve progression when bracing [4]. Previous studies have introduced quantitative flexibility tests in AIS where the applied force was transmitted through a flexible occipital-cervical junction by axial traction [6,7]; however, to our knowledge, no quantitative thoracolumbar test has been introduced.

A biomechanically consistent definition of spinal flexibility is a mathematical relation between the temporospatial changes in the spine and the force vector applied to generate this motion [1,6]. In this study, we introduce a controlled quantitative standing fulcrum side-bending test for the thoracolumbar spine (CSFS test) as a feasibility study to evaluate the forces needed when assessing spinal flexibility and whether the CSFS test would be a suitable tool for predicting the force required for spinal correction in AIS.

## 2. Materials and Methods

### 2.1. Design of the Study

The Regional Committee on Health Research Ethics approved the study (H-17034237). We obtained oral and written consent from the caregivers and subjects, including permission to screen medical records to test inclusion and exclusion criteria, and the study was conducted according to national guidelines and the Helsinki Declaration. The study was investigator-driven and supported internally by the Department of Orthopedics, Hvidovre Hospital, Denmark.

**Figure 1:**
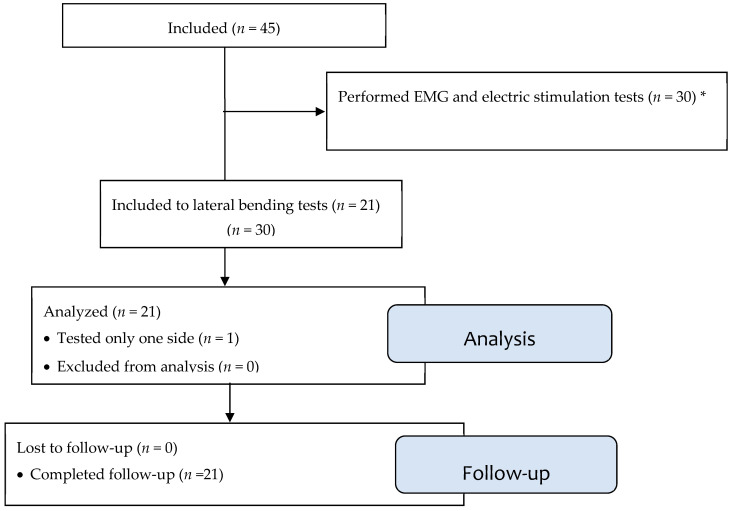
Timeline of the history of the subject’s participation; subjects were included in a study performing EMG and electric stimulation (*). In the interim, we closed this study, and 21 of the 45 subjects with AIS performed controlled lateral bending at the next clinical follow-up.

The study was a cross-sectional study of patients with AIS. They were included as subjects in a convenience sample from the outpatient clinic at our hospital. The inclusion criteria were adolescents diagnosed with mild to moderate scoliosis with a Cobb angle of 10° to 30°. The subjects were informed by an experienced paediatric orthopaedic surgeon in close consultation with the primary caregivers to ensure that the subjects were able to understand, cooperate, comply, and cope with the procedures of the study; if not, they were excluded. They were excluded if they participated in other studies/had treatments affecting their spine or spinal muscles and had spine-related operative treatments. After signing informed consent forms for both studies separately, they participated in previously published research on functional electric stimulation and electromyographic evaluation before then participating in this study of controlled bending at a subsequent follow-up (see Figure 1). The radiological examination substituted a planned clinical radiological follow-up to minimise the exposed radiation dose. The subjects wore shorts and a specially constructed t-shirt which was open in the back to expose the spinal column whilst covering the chest at the front during the examinations. Afterwards, the subjects were followed medium-term until maturity as part of the clinical follow-up. This was either when there was no risk of progression (maturity) with observation with or without interventions of physiotherapy or bracing or until surgery. We retrieved the baseline data of age, other spine-related diagnoses, and information on previous surgery and other spine-related medical treatment before or during monitoring the AIS, as well as the radiological evaluation of the Cobb angle for the subjects during monitoring until maturity/medium-term when available. Radiological examinations were typically performed every six months from detection to maturity according to clinical guidelines using a fluoroscopic technique described previously, creating a dynamic, cinematic sequence. The radiation dose of the bilateral examinations was one-fourth of a regular radiological examination for AIS [8,9].

### 2.2. The CSFS Test—Controlled Quantitative Standing Fulcrum Side-Bending Test

**Figure 2:**
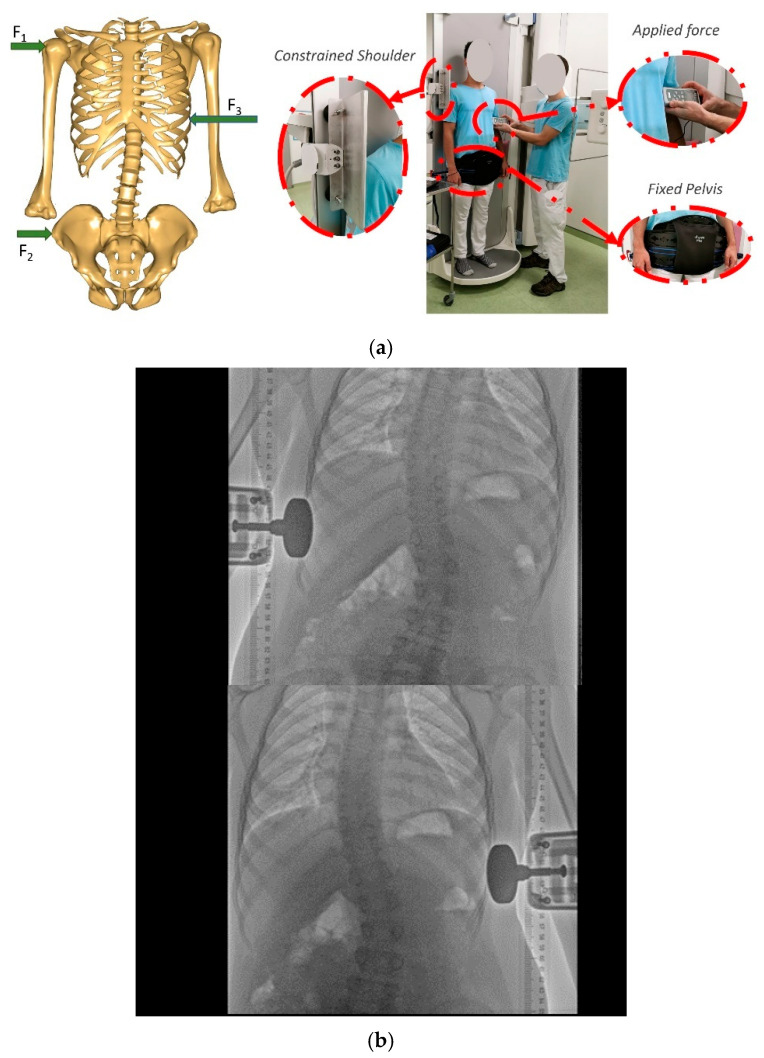
(**a**). Photographs and schematic depiction of the experimental force-and-deformation acquisition setup (top). We capture the low-dose radiographs of the subject’s torso while simultaneously increasing a controlled force (F3) on the ribs. This force was balanced by a force on the opposite shoulder (F1) obtained by resting the shoulder against a wall attached to the X-ray bed (see top-left inset) and a force on the pelvis (F2) produced by a fixing belt (see bottom-left inset). The three forces together resemble the three-point pressure principle of common scoliosis braces. Using force and torque equilibrium conditions, together with body measurements, we estimate the reaction forces on the shoulder and the pelvis. (**b**). Radiographs of a subject with force application from the two directions (from left to right) (below).

For the CSFS test, we developed an experimental setup that allowed for lateral bending of the torso in stance by inducing a quantified lateral force while capturing the dynamic changes in the spinal deformations under fluoroscopic exposure. To mimic the three-point pressure principle, we applied a force on the ribs on one side of the subject while keeping the pelvis and the opposite shoulder fixed (Figure 2a).

The rib force was applied through a Sauter FH500 force gauge (KERN & SOHN GmbH, Balingen, Germany) (Range: 0–500 N. precision: 0.005 of max) [10], thus providing a measurement of the applied force continuously in stance, and with the pelvis fixed by a belt, whilst the subject was resting the shoulder on a rigid wall attached to the X-ray bed. The surface of the bed was smooth, ensuring minimal friction. The back of the subject was directly adjacent to the X-ray bed to avoid torsion of the torso during lateral bending. The three-point pressures in the frontal plane of the setup were reactions F1 on the shoulder, F2 on the pelvis, and the applied force, F3, on the ribs. F3 was ramped up while recording a series of radiographs in one capture. The setup was repeated symmetrically on both sides. Subjects were instructed to give notice if the force became uncomfortable. Force application was performed by the same operator in all trials. The resulting radiograph sequence is illustrated in Figure 2b.

### 2.3. Data Processing and Statistical Analysis

Force data time series were automatically registered on file from the force gauge, and Cobb angles were recorded frame-by-frame from the X-ray sequences. The force signal was resampled to the frame rate of the X-rays, forces were charted against the Cobb angles, and linear regression was performed to determine stiffness and correlations between applied forces and Cobb angle changes.

To test for potential relationships, the *p* and the *R*^2^ values of all trials were evaluated statistically. *p*-values of ≤0.05 were considered statistically significant, and we applied Bonferroni corrections to our analyses when appropriate. Assessment for the variance of the residuals/heteroscedasticity was evaluated by the histograms of the residuals and normal probability plot. All tests were performed using IBM SPSS Statistics, Version 25 (IBM, Richmond, VA, USA).

## 3. Results

Forty-five patients with AIS and their caregivers were included as subjects after screening medical records against inclusion and exclusion criteria. The included subjects were scheduled for a CSFS test at their next planned radiological follow-up. Figure 1 shows a flow chart of the subject’s participation and exclusion history. We retrieved the baseline data of the initial Cobb angles and the Cobb angles at evaluation (reflecting the severity of curvature at diagnosis and progression), curve types (e.g., thoracic or lumbar), and MRI performances. We also gathered the patients’ ages during evaluation, the monitoring lengths (from diagnosis to evaluation), progression statuses at the final evaluations at the radiological follow-ups, medium-term, and information on spine-related treatments, if any. This is described in Table 1.

### The CSFS Test

**Figure 3:**
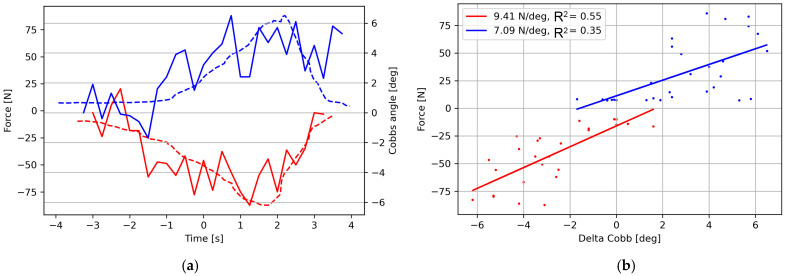
Examples of time series of the applied force, the Cobb angles (**a**), and the resulting stiffness assessments and correlations (**b**). Blue colour designates force from the right-hand side, and red is from the left-hand side.

Twenty-one patients were included as subjects for the CSFS test. All subjects performed the test adequately and were included in the analysis, although one subject underwent testing from one side to minimise radiation exposure. The gender ratio (female:male) was 15:6. The average initial mean Cobb angle was 11.8° (SD: 5.5°). They were examined after a mean of 2.2 years (SD: 2.15) after the diagnosis of AIS. The average age at examination was 13.3 years (SD: 2.4) with a mean Cobb angle of 12.0° (range: 0–26°, SD: 7.1°). Figure 3 illustrates selected examples of the force–time curves and the resulting correlations between applied forces and Cobb angles. The stiffnesses in units of force per change in Cobb angle and the R^2^ value for linear regression analyses for all participants are shown in Table 2.

**Figure 4:**
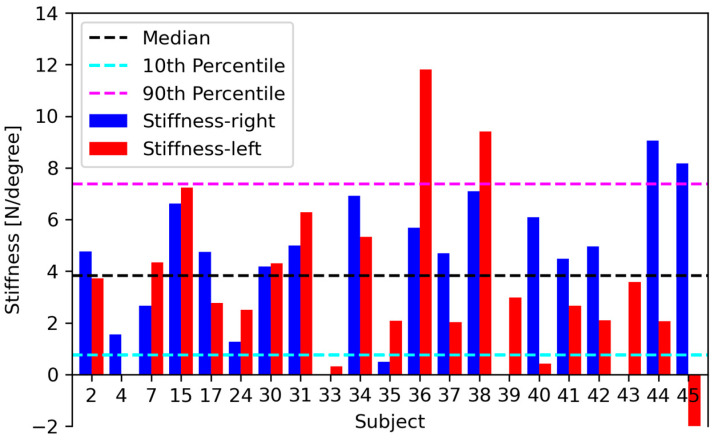
Stiffnesses for individual subjects with median in the N/degree and the 10th and 90th percentiles—right (blue bar) and stiffness—left (red bar). X-axis with subject numbers.

Table 3 shows the median values, the 10th and 90th percentiles of stiffnesses in the N/degree of the Cobb angle, and, for both sides, the *R*^2^ values, maximal applied force, and overall change in the Cobb angle during the CSFS test. The average R^2^-value is 0.35. Figure 4 illustrates the median stiffnesses for individual subjects from the left and right side.

There were no significant relationships with the median-term clinical outcome of either progression/regression or stationary curves, various Cobb angle measurements (i.e., initially and at the CSFS test), and the other experimental parameters in Table 1 and Supplementary Materials Tables S1 and S2. Selected radiological captures and videos of the cinematic sequences, the force–time curves, the time/force, and the change in the Cobb angle curve for 2–45 subjects are also included in the Supplementary Materials.

## 4. Discussion

This study has investigated the feasibility of a controlled quantitative standing fulcrum side-bending method in AIS. With the CSFS test, we could evaluate and correlate the applied force to changes in the Cobb angle, facilitating the assessment of necessary forces to obtain a given correction, typically by bracing.

The regressions from which the stiffnesses are computed follow from an implicit hypothesis of linear stiffness. However, the average R^2^ value of all regressions is only 0.35, so it does not confirm the existence of such a linear relationship. A closer investigation of the raw data (Figure 3) reveals that the Cobb angle assessments from the radiographs are subject to significant noise, likely due to the difficulty of reliably reading small angle differences from blurry images. Without this noise, the R^2^ values would likely be significantly improved.

We have presented all data, including outliers and cases that may be erroneous, because this is a feasibility study designed to evaluate an experimental technique. Figure 4 shows that the results differ much between subjects and sides. Of particular interest are subjects 33, 35, 40 and 45, all presenting stiffness below the 10th percentile, and negative stiffness in one case. All these cases are characterised by R^2^ values below 0.05, so the results designate inconsistent measurements rather than credible stiffnesses.

Subjects 36 and 38 in the left direction and 44 and 45 in the right direction present stiffnesses beyond the 90th percentile. These outliers cannot be disregarded due to small R2 values, but the radiographs reveal that the piston applying the force was misaligned with the apex of the curve in these cases. This diminishes the effect of the force on the Cobb angle and translates to high stiffness.

The outliers indicate that the experimental method is not entirely robust and that additional refinement of the protocol remains to ensure the validity and reliability of the data. Aligning the piston with the apex of the curve appears to be a simple improvement, but the misalignment only becomes obvious after the experiment when the radiographs have been taken.

There are notable positive associations between spinal flexibility and the efficiency of bracing, as indicated by flexibility testing and in-brace correction rates [3]. Research indicates that spine flexibility, assessed by several radiography and imaging methods, is a significant predictor of the first in-brace Cobb angle correction. Several correlations were identified, including r = 0.65 and r = 0.74, indicating moderate to high connections between the flexibility rate in supine radiographs and in-brace [11,12]. Nonetheless, not all studies concurred on the importance of spinal flexibility in forecasting brace effects [12]. There seems to be an association between spinal flexibility and brace treatment outcomes; nevertheless, the predictive value of flexibility may differ according to patient-specific characteristics and curve attributes [3]. If the relationship between force and Cobb angle correction by bracing can be reliably detected and the method has potential clinical applications, it allows for the estimation of the resulting skin pressures in the clinical scenario of brace application for smaller curves. Excessive pressure on the skin from braces can impede blood circulation, resulting in pressure ulcers or pain for the patient, with acceptable pressure limits being generally below 20–40 mmHg [13]. In a typical case of stiffness, which, according to the data, could be a 4 N/degree change in Cobb angle, a correction of, for example, 20 degrees would require a constant bracing force of 80 N. If this is assumed to be distributed between the brace and skin over an area of 100 × 100 mm^2^ of the brace/skin interface, the resulting pressure would be 800 Pa = 6.0 mmHg, i.e., well within the acceptable range. However, recorded stiffnesses range significantly, and so do required corrections, so the results may be different for any measured patient. The CSFS measurement therefore seems to be useful for estimating the feasibility of bracing curves with a measured stiffness to a desired correction within safe limits of skin irritation, tissue damage, and available corrective force from the brace.

In the clinical scenario of spinal surgery for larger curves, the bone–screw interface can be exposed to forces overloading tissues or implants. Excessive forces can lead to “ploughing”, where screws shift within the bone, risking complications like screw pull-out or failed spinal fusion. The forces exerted on the spine and measured at the pedicle screws during anterior fusion scoliosis surgery, when measured in vivo, have been reported to be 540 N (SD 230 N; range 88 N–1019 N) [14]. The ranges of our stiffness measurements indicate that this may vary significantly between patients. The translation of CSFS stiffnesses to pedicle screw forces can be accomplished with a finite element analysis, leading to guidelines for safe scoliosis spinal fusion procedures based on a CSFS test of the patient [15].

Changes in flexibility when using the supine traction method [1], compared with the lateral bending method, suggests that the patient’s muscle contraction and co-contractions have a significant impact on the correction of the curve during the assessment of spinal flexibility. The benefit of the CSFS test would be to eliminate voluntary and involuntary muscle resistance more than the other tests since we apply the force without subject involvement, as described below. In general, the corrective loads found in this study cannot be compared with results using other loading types (for instance, axial traction [6,16]) because the bending moments experienced by the spine depend entirely on the method of load application. Furthermore, the loads are transferred to the spine via the ribs and thorax, whose stiffnesses also contribute to the result. Thus, measured spine stiffnesses are specific to the applied measurement method.

The latter confirms the tenets of other flexibility studies, which might not be surprising since our technique combines the merits of the supine side-bending method (performing supine side bending in our study), the fulcrum-bending method (passively hinged), traction (push), the push-prone method (push by force-lever), and the suspension method (subjects strapped to the wall) [1,15]. The flexibility evaluation method of this study is a simplification of the complex biomechanics of the spine. Spinal flexibility is a measure of spinal mechanics which captures the overall behaviour of the structure (i.e., how much it deforms under a load) rather than the intrinsic mechanical properties of the spinal components and their various tissues. The measured flexibility reflects the combined effect of various internal structures, such as muscles, ligaments, annuli, and discs, and it is currently not possible to isolate the contribution of each of these structures to overall flexibility. Moreover, we have the biomechanics of the ribs and costovertebral joints added to our flexibility evaluation. This limitation is not unique to this study and is shared by traditional clinical assessment techniques. Additionally, the level of muscle contraction can significantly impact overall stiffness, and the CSFS method is an attempt to reduce this part.

It has previously been suggested that, to validate the accuracy of the flexibility measurement technique, it would be necessary to obtain intra-operative stiffness measurements from the same patients [7]. However, this validation phase would require significant effort to obtain accurate intra-operative stiffness measurements, which is beyond the scope of the present study and not suited for our subjects with small-curve AIS; additionally, it not feasible since the CSFS test is performed in stance.

In future studies, curve magnitude and location are important considerations when using the CSFS test to assess spinal flexibility. At this point, the CSFS setup should be considered to be formed for experimental research on AIS until further studies have characterised the method for clinical feasibility.

The force in the CSFS test is applied to the trunk manually and gradually, as seen in the force–time curve in the Figures S2 and S3 in the Supplementary Materials. This ensures the comfort and safety of the subject, and it enables examination of the spatial changes in the full dynamic spinal movement continuously using fluoroscopy, whereas other quantitative methods evaluate the spinal movement at the end-position of the force application using bi-planar images [6,7]. The raw results presented in Figure 3 show that Cobb angle assessment from the radiographs is subject to noise and uncertainty, indicating that calculation of the stiffness over a range of force/Cobb angle values, as in the present method, benefits the precision. However, this should be seen as the first step to quantifying spinal stiffness and flexibility. After refinement of this method, as suggested, further evaluation using other flexibility methods as a control and including larger curves is warranted before potentially being implemented directly for patient-specific bracing or for surgical interventions using in silico simulation methods in treatment planning and in general for developing novel strategies for correcting spinal deformities [7,17,18,19,20].

We were unable to determine whether the data could be utilised as a prognosticator for treatment efficacy since 19/21 of our curves were stationary or regressed and only two progressed to candidates for bracing. Moreover, the position of the piston was fixed, making the method infeasible for testing certain primary curves, such as high thoracic or low lumbar, and subsequently not appropriate for evaluation of the curve progression of these curves. Moreover, the force application was performed manually due to the concern and comfort of the subject; thus, it was not performed with a consistent proportional increase but more in an inconsistent incremental manner in accordance with the response of displacement/lateral bending during testing, and this was decided at the discretion of the examiner, as was deciding when to end the test. Future studies also would benefit from monitoring pain effects and other sensory/motor disturbances induced by the CSFS test. The CSFS test might only be feasible for thoracolumbar curves, and future studies could examine applicability and use for prognostication, which, as mentioned, was unfeasible when testing small curves and examining larger curves for estimating stiffness for deformity correction, i.e., brace application or spinal surgery. The assessment of the flexibility using the CSFS test has been utilised for the fundamental mechanical characterisation of the deformity [15]. The alignment of the piston with the apex of the curve was insufficient, partially because of inflexible adjustment options being unable to visualise the alignment to the deformity whilst performing the test. The piston was generally applied at the thoracolumbar level, and high thoracic curves were out of reach. For future studies, one should be aware of this, since this problem can be solved technically; however, in the current setup, the CFCS method seems to be purposeful for low thoracic, thoracolumbar, and high-lumbar curves. However, this should be seen as the first step to quantify spinal stiffness and flexibility and should be potentially implemented directly for patient-specific bracing or for surgical interventions using in silico simulation methods in treatment planning and in general for developing novel strategies for correcting spinal deformities [5,15,16,17,18].

In this study, we tested smaller curves. Other flexibility tests are suited for moderate to severe curves and have only been tested for those. Their precision for smaller curves, as investigated in this study, has yet to be determined [6]. The radiological bilateral procedure includes one-fourth of the radiation dose of a regular posteroanterior AIS radiograph [9], but we could not justify performing double examinations and performing other flexibility tests on the subjects to evaluate the precision and accuracy of the CSFS test due to excess radiation doses.

When selecting an appropriate method for assessing spinal flexibility and predicting treatment outcomes, two crucial parameters to consider are the magnitude and location of the curve [1]. Various flexibility measurement methods reveal significant subtleties, particularly in terms of reliability over curve severity. The supine method exhibits 25% spinal flexibility, which is often inferior to dynamic techniques such as lateral bending or traction. The lateral bending method demonstrated that moderate curves exhibit greater flexibility than severe curves, with thoracolumbar/lumbar curves displaying superior flexibility compared to thoracic curves. The fulcrum-bending techniques showed increased flexibility for thoracolumbar/lumbar curves (53% to 83%) in contrast to thoracic curves (45% to 74%). Use of the manual correction technique demonstrates that mild curves are more flexible than severe ones, exhibiting flexibilities of between 30 and 40% for thoracic curves and 30–50% for thoracolumbar/lumbar curves, and when using the traction approach as, i.e., standing suspension, demonstrates flexibility of 40% for thoracic curves and 45% for thoracolumbar/lumbar curves and when combining traction and manual correction techniques demonstrated flexibility of 55% for thoracic curves and 65% for thoracolumbar/lumbar curves. In general, thoracolumbar/lumbar curves demonstrate superior flexibility compared to thoracic curves across several methodologies, but moderate curves exhibit greater flexibility than severe curves. For patients with severe curves, the traction method should be selected, while the lateral bending method is more suitable for those with moderate curves. To assess thoracic curves’ curve flexibility, the fulcrum-bending method is recommended, whereas, for higher thoracic or thoracolumbar/lumbar curves, the supine-with-lateral bending method is more appropriate.

In this study, the subjects had similar ages but an over-representation of the female sex and variance in curve morphology and level, as we included subjects as a convenience sample, which was thus reflected in the large range of stiffness, even disregarding the outliers. Due to our limited number of 21 subjects and the heterogeneity of demographics and curves, we were unable to perform meaningful analyses related to these parameters. Previous studies have included from 5 to 70 subjects; thus. the number of included subjects must be considered a study limitation [1]. The weak correlation between spinal stiffness and applied force might also be due to the general variation in Cobb angle measurement and certainly during lateral bending [21]. Moreover, the Cobb angle was dispersed, but in the lower spectrum, and subjects varied regarding the time from AIS ‘onset’ to time for testing, as well as time to testing to maturity, potentially affecting the flexibility of the spine. Since the focus was on small-curve AIS, some subjects regressed to almost straight spines after inclusion and when examined with the CSFS test. Thus, there was an overall decreased risk of progression amongst the subjects. We did not detect an interrelation between stiffness and parameters for the Cobb angle. However, this might have been obscured by the confounding factors.

## 5. Conclusions

In conclusion, the CSFS method is feasible for quantifying the applied force and tracking the temporospatial changes in the spine, and we are able to measure the continuous change in Cobb angle and externally applied forces during lateral bending. The method requires refinement before clinical application but potentially can involve a quantitative bending in the thoracolumbar spine and a tool estimating skin pressure in-brace fitting.

## Figures and Tables

**Table 1:** Patient characteristics for the specific subjects.

Pt Number	Initial Cobb’s Angle	Curve Type	MRI	Cobb’s Angle at Evaluation	Age at Examination (Years)	Time Diagnosis to Evaluation (Months)	End Evaluation
2	11.00	sin TL konx	-	24.00	12.43	71.97	1
4	7.00	T dex konc	-	17.00	13.04	56.48	0
7	26.00	S dex TL	Ia	21.00	18.68	12.59	0
15	4.00	S dex konx	arkolyse l5	13.00	15.64	49.22	0
17	10.00	high T sin konx	-	3.00	11.81	12.99	0
24	0.00		-	0.00	12.04	−10.65	0
30	12.00	high T dex konx	Ia	8.00	14.34	30.35	0
31	11.00	L sin konx	-	11.00	15.31	0.00	0
33	14.00	short TL sin	-	8.00	9.77	84.23	0
34	11.00	L sin konx	-	14.00	14.65	63.06	0
35	18.00	high T dex	-	10.00	11.73	3.81	0
36	10.00	high T dex	-	10.00	13.61	6.02	0
37	10.00	L sin konx	Ia	10.00	12.21	28.54	0
38	13.00	L sin konx	-	11.00	15.05	46.22	0
39	11.00	TL dex konc	-	10.00	9.52	23.80	0
40	18.00	L dex konx	Ia	26.00	16.19	4.77	0
41	18.00	S T dex	Ia	20.00	9.67	5.75	1
42	10.00	C TL dex	-	10.00	12.88	7.66	0
43	10.00	L dex konx		15.00	14.76	18.94	0
44	7.00	L sin konx		5.00	10.21	26.27	0
45	16.00	C TL		4.10	14.81	18.71	0

Pt—Patient, sin—left, dex—right, TL—thoracolumbar, T—thoracic, L—lumbar, S—S-shaped, C—C shaped, konc—concave, konx—convex, MRI—Magnetic Resonance Imaging, Ia—no pathological intramedullary findings on MRI End evaluation 0 = curve unchanged or regressed, End evaluation 1 = curve progressed to brace wearing.

**Table 2:** Stiffness (force of correction of scoliosis per degree of Cobb angle) in N/degree, Stiffness -Right and Stiffness -Left in N/degree of Cobb angle, R-square values for the regression analyses for the right (R) and left (L) side and calculated force of Maximal applied force (average of left and right).

Subject	Stiffness-Right	Stiffness-Left	R^2^-Right	R^2^-Left	Force Max
2	4.77	3.73	0.91	0.60	101.89
4	2.66	4.34	0.60	0.68	73.58
7	6.62	7.23	0.67	0.76	90.01
15	4.74	2.77	0.90	0.56	11.28
17	1.28	2.50	0.18	0.48	-
24	4.18	4.31	0.37	0.75	33.93
30	5.00	6.29	0.56	0.55	62.08
31	6.91	5.33	0.76	0.69	85.72
33	0.49	2.09	0.10	0.51	12.89
34	5.69	11.81	0.55	0.61	87.50
35	4.69	2.02	0.52	0.14	33.52
36	7.09	9.41	0.59	0.74	90.75
37	6.08	0.41	0.57	0.04	84.43
38	4.49	2.67	0.71	0.26	71.58
39	4.95	2.10	0.86	0.54	35.25
40	0.02	3.58	0.00	0.85	27.03
41	9.06	2.06	0.82	0.65	27.79
42	8.17	−2.12	0.42	−0.22	12.40

**Table 3:** The median, 25 and 75 percentile of Stiffness (force of correction of scoliosis per degree of Cobb angle) in N/degree, Stiffness-Right and Stiffness-Left in N/degree, R-square values for the regression analyses for the left (L) and right (R) side, the maximal applied Force left and right in N, and overall change in Cobb angle left and right in degree.

	Stiffness	Stiffness-Right	Stiffness-Left	R-Right	R-Left	Force Max Left	Diff Cobbs Left	Force Max Right	Diff Cobbs Right
Median	4.37	4.86	3.18	0.58	0.58	75.60	18.50	74.20	14.10
25 Percentile	2.20	3.80	2.08	0.41	0.42	55.20	12.70	56.60	8.25
75 Percentile	6.24	3.80	2.08	0.78	0.70	101.10	21.65	88.00	23.55

## Data Availability

For further interest, inquiries for data can be sent to the corresponding author.

## Supplementary Materials

The following supporting information can be downloaded at: https://www.mdpi.com/article/10.3390/jcm13247809/s1. Figure S1. Radiological image of the controlled bending; Figure S2. Time force curve for 2–45 subject; Figure S3. Time/force and change in Cobb angle curve for 2–45 subjects; Videos S1–S9 Examples of bending tests; Table S1: The measured Cobb angle before and after applied maximal force, non-normalized and normalized for the specific subjects.; Table S2. The applied maximal force and time from initial force application to maximal applied force for the specific subjects.
