# Supplementary material for: A Feasibility Study of a Controlled Standing Fulcrum Side-Bending Test in Adolescent Idiopathic Scoliosis
Source: J Clin Med. 2024 Dec 20;13(24):7809. doi: 10.3390/jcm13247809 (PMC11676229; doi:10.3390/jcm13247809)
Supplement: Supplementary file 1 [file jcm-13-07809-s001.zip › jcm-3328261-supplementary/jcm-3328261-supplementary.pdf]

Supplementum The CSFS-test - Controlled quantitative standing fulcrum side-bending test

Figure S1. Radiological image of the controlled bending.

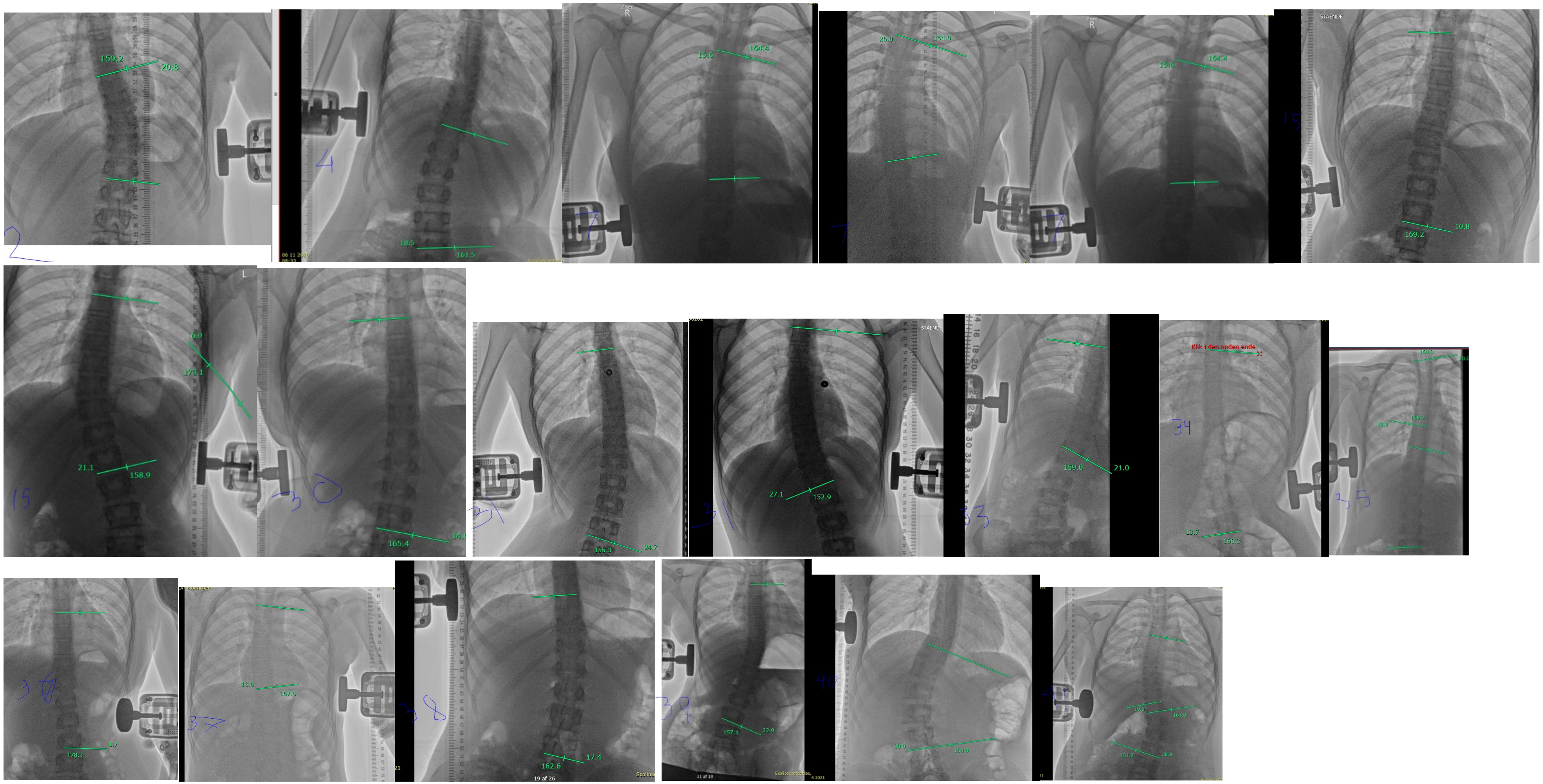

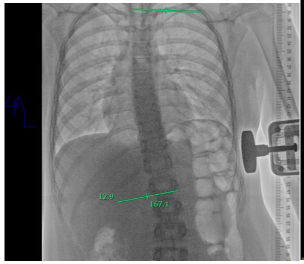
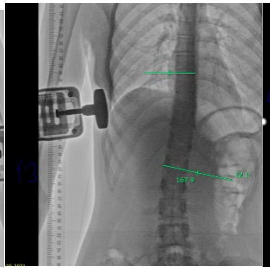
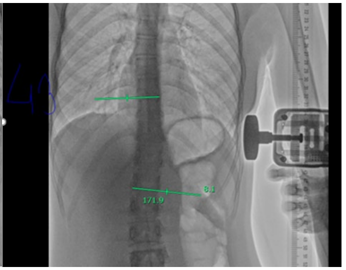

Figure S2. Time force curve for 2-45 subjects

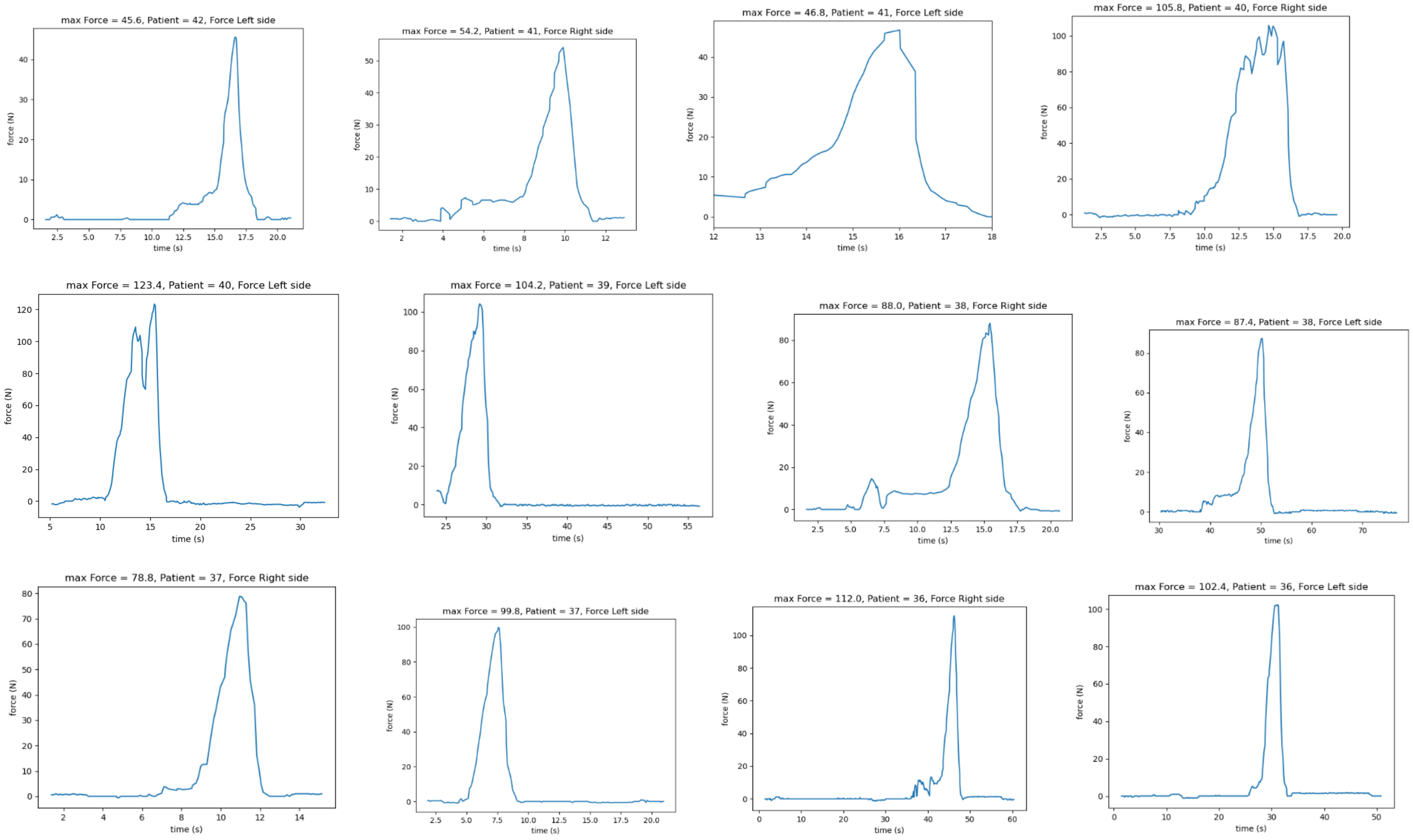

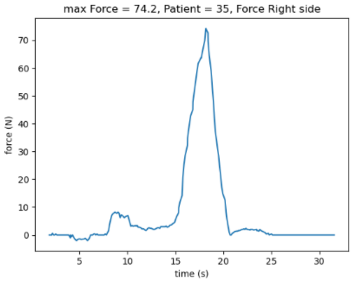
max Force = 74.2, Patient = 35, Force Right side
force (N)
time (s)

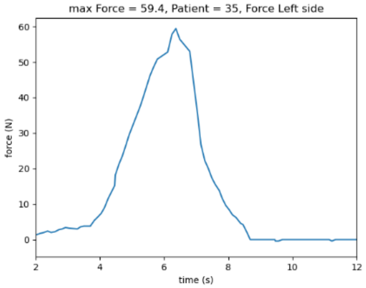
max Force = 59.4, Patient = 35, Force Left side
force (N)
time (s)

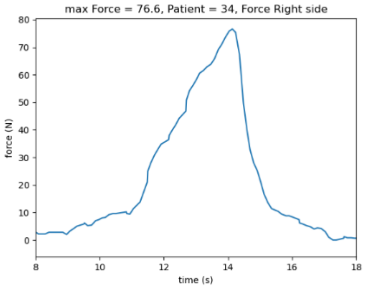
max Force = 76.6, Patient = 34, Force Right side
force (N)
time (s)

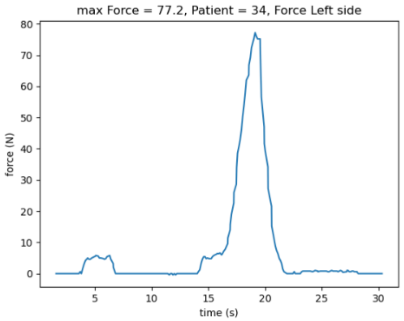
max Force = 77.2, Patient = 34, Force Left side
force (N)
time (s)

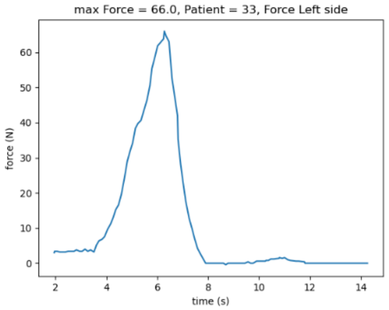
max Force = 66.0, Patient = 33, Force Left side
force (N)
time (s)

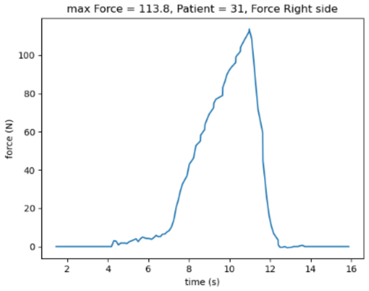
max Force = 113.8, Patient = 31, Force Right side
force (N)
time (s)

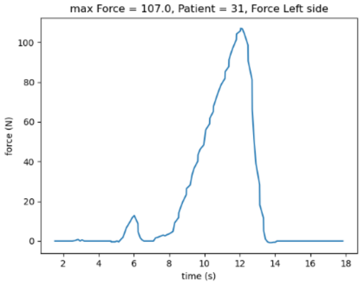
max Force = 107.0, Patient = 31, Force Left side
force (N)
time (s)

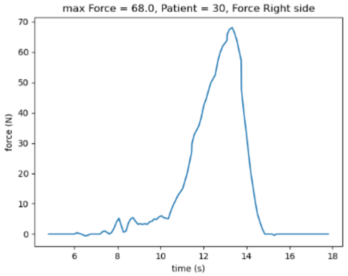
max Force = 68.0, Patient = 30, Force Right side
force (N)
time (s)

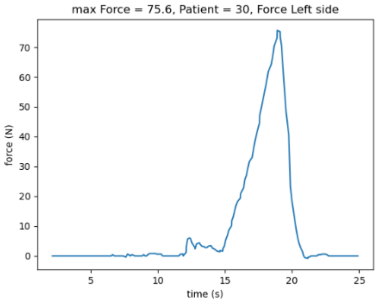
max Force = 75.6, Patient = 30, Force Left side
force (N)
time (s)

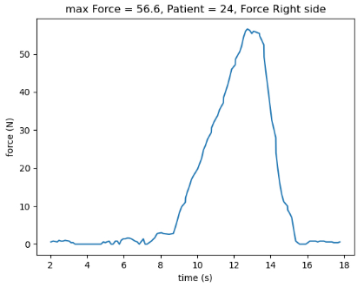
max Force = 56.6, Patient = 24, Force Right side
force (N)
time (s)

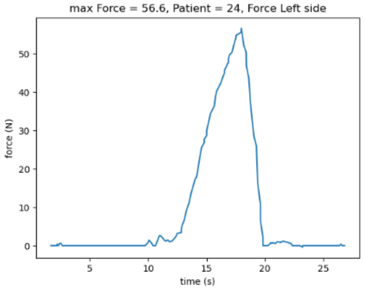
max Force = 56.6, Patient = 24, Force Left side
force (N)
time (s)

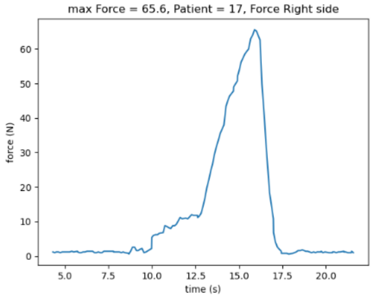
max Force = 65.6, Patient = 17, Force Right side
force (N)
time (s)

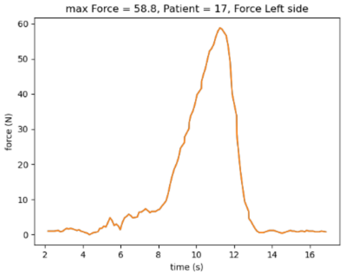
max Force = 58.8, Patient = 17, Force Left side
force (N)
time (s)

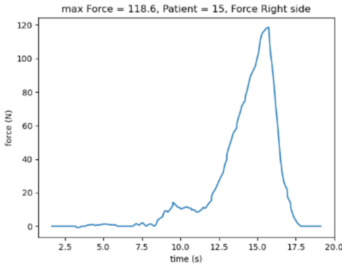
max Force = 118.6, Patient = 15, Force Right side
force (N)
time (s)

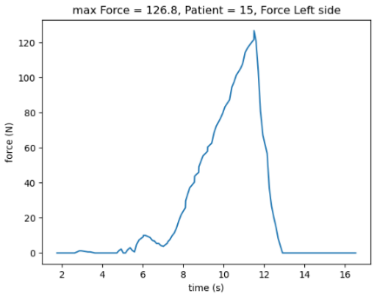
max Force = 126.8, Patient = 15, Force Left side
force (N)
time (s)

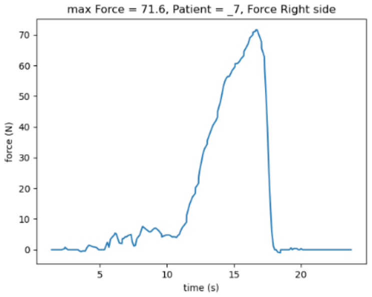
max Force = 71.6, Patient = _7, Force Right side
force (N)
time (s)

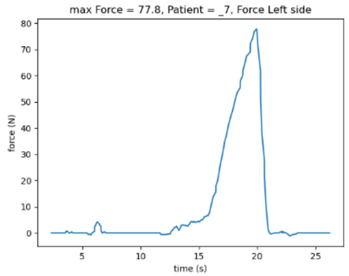
max Force = 77.8, Patient = _7, Force Left side
force (N)
time (s)

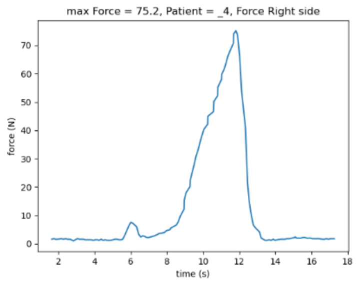
max Force = 75.2, Patient = _4, Force Right side
force (N)
time (s)

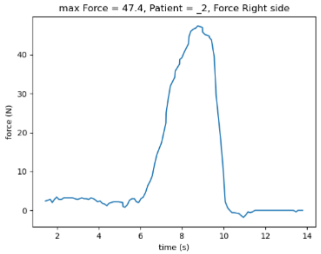
max Force = 47.4, Patient = _2, Force Right side
force (N)
time (s)

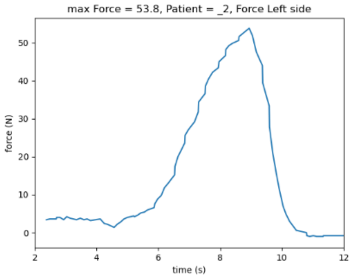
max Force = 53.8, Patient = _2, Force Left side
force (N)
time (s)

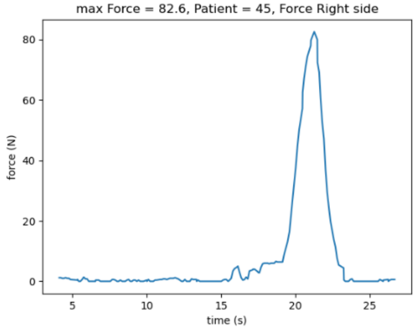
max Force = 82.6, Patient = 45, Force Right side
force (N)
time (s)

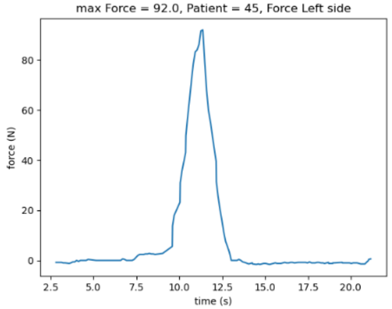
max Force = 92.0, Patient = 45, Force Left side
force (N)
time (s)

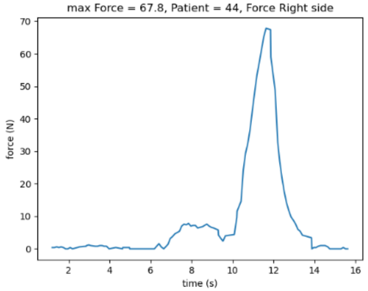
max Force = 67.8, Patient = 44, Force Right side
force (N)
time (s)

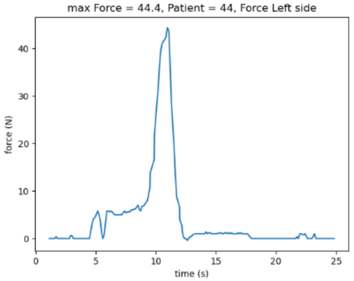
max Force = 44.4, Patient = 44, Force Left side
force (N)
time (s)

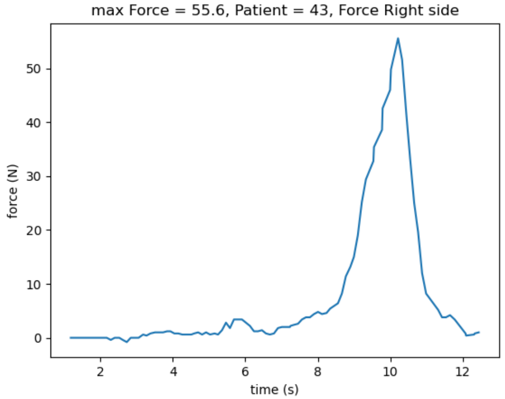
max Force = 55.6, Patient = 43, Force Right side
force (N)
time (s)

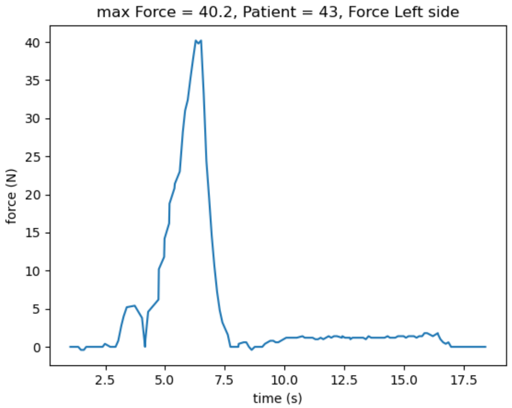
max Force = 40.2, Patient = 43, Force Left side
force (N)
time (s)

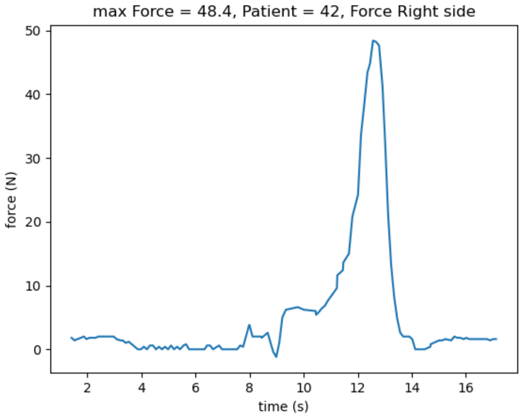
max Force = 48.4, Patient = 42, Force Right side
force (N)
time (s)

Figure S3. Time/force and change in Cobb angle curve for 2-45 subjects.

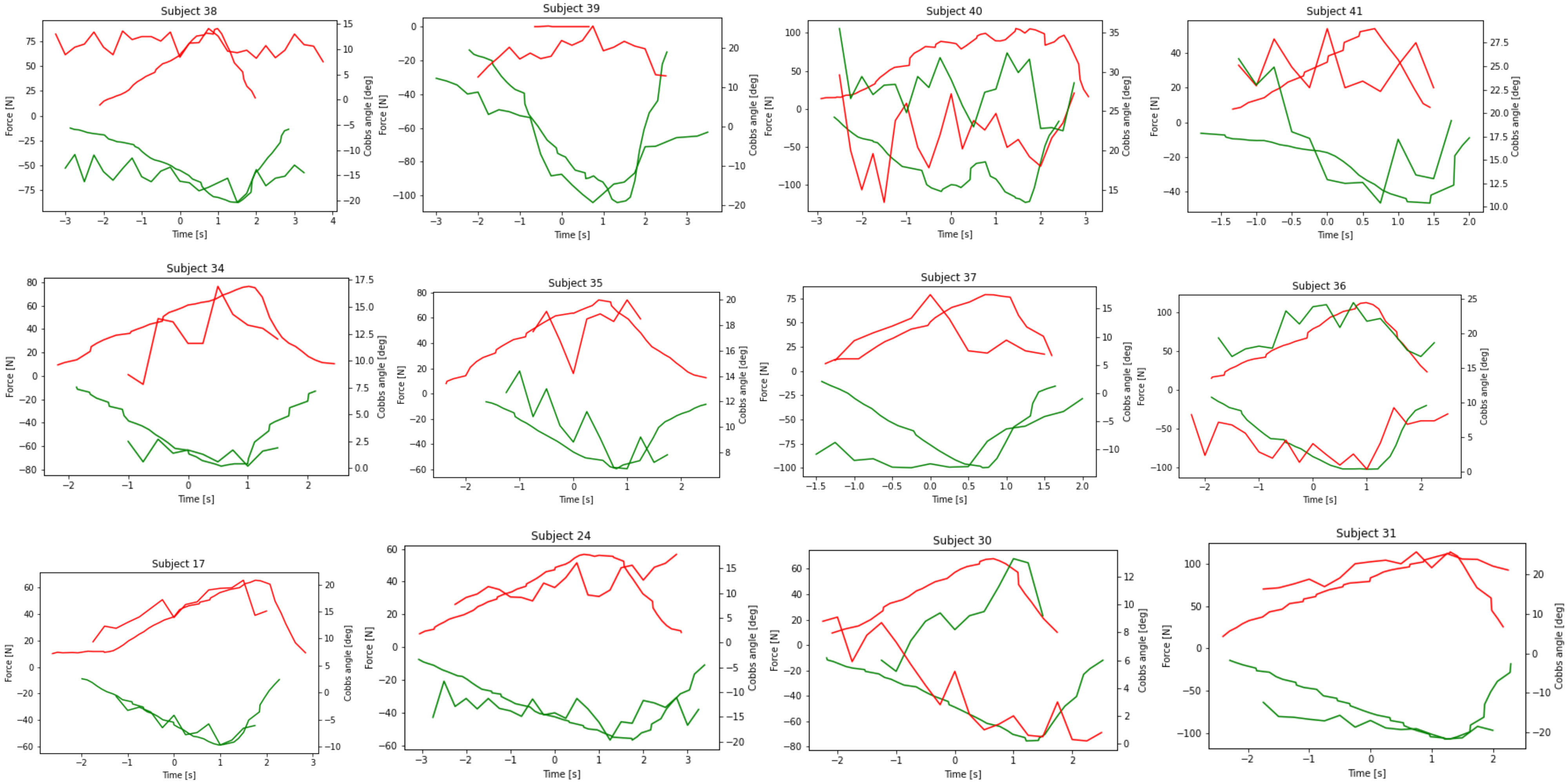

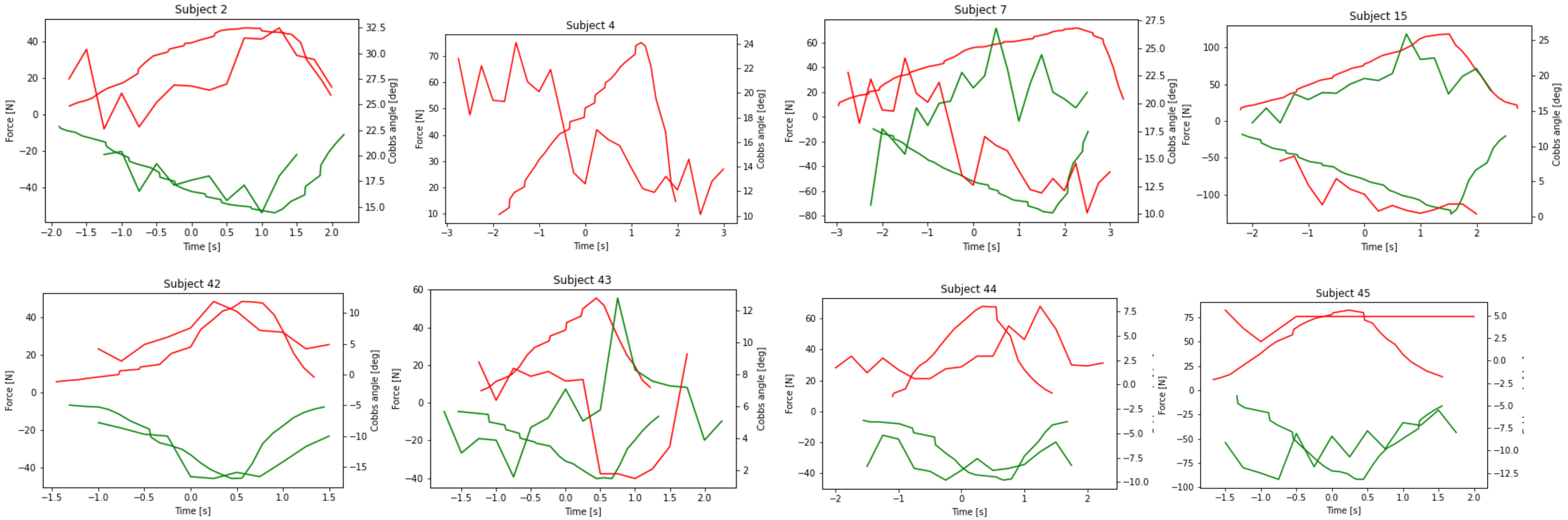

Subject 2
Subject 4
Subject 7
Subject 15
Subject 42
Subject 43
Subject 44
Subject 45
Force [N]
Cobbs angle [deg]
Time [s]

Pt number	Cobb angle before CB - L	Cobb angle at max CB - L	Cobb angle before CB - R	Cobb angle at max CB - R	Change in Cobb angle - NL	Change in Cobb angle - NR	change L	change R
2	24,3	24,4	35,8	32,9	0,1	0,1	0,1	-2,9
4	16,4	17,0			0,6		0,6	
7	21,8	21,9	15,6	21,0	0,1	0,3	0,1	5,4
15	24,2	24,3	0,0	6,6	0,1	0,5	0,1	6,6
17	19,1	19,4	0,0	8,3	0,3	0,9	0,3	8,3
24	14,7	15,0	8,7	15,4	0,3	0,6	0,3	6,7
30	10,1	10,6	0,0	13,9	0,5	2,6	0,5	13,9
31	20,1	20,1	15,3	21,0	0,0	0,3	0,0	5,7
33	11,3	12,0	24,1	26,6	0,7	0,1	0,7	2,5
34	17,2	17,5	3,2	10,9	0,3	0,7	0,3	7,7
35	24,1	24,1	7,1	18,3	0,0	0,7	0,0	11,2
36	18,0	18,3	2,7	7,0	0,3	0,4	0,3	4,3
37	8,0	10,0	12,1	15,0	2,0	0,3	2,0	2,9
38	12,9	13,3	14,4	19,2	0,4	0,3	0,4	4,8
39	21,3	21,6	-10,6	22,2	0,3	6,0	0,3	32,8
40	22,5	22,6	16,6	19,6	0,1	0,2	0,1	3,0
41	25,6	25,8	20,0	27,0	0,2	0,3	0,2	7,0
42	20,3	20,4	17,1	21,4	0,1	0,2	0,1	4,3
43	20,1	20,5	10,2	14,4	0,4	0,3	0,4	4,2
44	18,1	18,7	7,6	10,3	0,6	0,2	0,6	2,7
45	7,0	7,3	10,2	-6,1	0,3	1,9	0,3	-16,3

Table S1. The measured Cobb angle before and after applied maximal force, non-normalized and normalized for the specific subjects. Pt – Patient, CB – controlled bending, L – left, R – right, NL – normalized left, NR- normalized right and change – ratio between Cobb angle before and after.

Pt number	force max left	time left	ratio force /time left	force max right	time right	ratio force /time right
2	53,80	3,00	17,93	47,40	3,00	15,80
4	75,20	3,00	25,07	77,80	3,00	25,93
7	77,80	5,00	15,56	71,60	7,00	10,23
15	126,80	4,00	31,70	118,60	3,50	33,89
17	58,80	4,00	14,70	65,60	3,50	18,74
24	56,60	4,50	12,58	56,60	4,00	14,15
30	75,60	4,00	18,90	68,00	3,50	19,43
31	107,00	4,00	26,75	113,80	4,00	28,45
33	66,00	2,20	30,00			
34	77,20	4,30	17,95	76,60	2,20	34,82
35	59,40	2,20	27,00	74,20	3,00	24,73
36	102,40	2,50	40,96	112,00	3,00	37,33
37	99,80	2,50	39,92	78,80	2,50	31,52
38	87,50	3,00	29,17	88,00	2,80	31,43
39	104,20	4,00	26,05			
40	123,40	5,00	24,68	105,80	6,00	17,63
41	46,80	2,00	23,40	54,20	2,00	27,10
42	46,50	2,50	18,60	48,40	2,00	24,20
43	40,20	2,00	20,10	55,60	2,00	27,80
44	44,40	1,50	29,60	67,80	2,00	33,90
45	92,00	2,50	36,80	82,60	2,30	35,91

Table S2. The applied maximal force and time from initial force application to maximal applied force for the specific subjects. Pt – Patient, force max left – maximal applied force on the left side, time left – time from initial force application to maximal applied force on the left side, ratio force/time left – the ratio between the maximal applied force divided by applied on the left side, force max right – maximal applied force on the right side, time right – time from initial force application to maximal applied force on the right side, ratio force/time right – the ratio between the maximal applied force divided by applied on the right side.
